# Current approaches to prescription and optimization of peritoneal dialysis: a practical review

**DOI:** 10.1590/2175-8239-JBN-2025-0288en

**Published:** 2026-06-12

**Authors:** Thyago Proença de Moraes, Caio Pellizzari, Paulo Ricardo Gessolo Lins, Viviane Calice-Silva

**Affiliations:** 1 Pontifícia Universidade Católica do Paraná, Curitiba, PR, Brazil.; 2 Universidade Federal de São Paulo, São Paulo, SP, Brazil.; 3 Fundação Pró-rim, Departamento de Pesquisa, Joinville, SC, Brazil.; 4 Universidade da Região de Joinville, Faculdade de Medicina, Joinville, SC, Brazil.

**Keywords:** Prescriptions, Residual Kidney Function, Ultrafiltration, Icodextrin, Automated Peritoneal Dialysis, Peritoneal Dialysis, Continuous Ambulatory

## Abstract

Peritoneal dialysis (PD) is an established form of kidney replacement therapy that offers patients the possibility of homebased treatment with significant flexibility and autonomy. Despite its advantages, many physicians in training and those just starting their clinical practice feel uncertain when prescribing PD, given the technical aspects, individualized adjustments, and the variety of modalities available. This manuscript provides a practical and didactic overview of how to prescribe PD, focusing on both continuous ambulatory peritoneal dialysis (CAPD) and automated peritoneal dialysis (APD). The discussion begins with the fundamental principles of solute and fluid transport, followed by step-by-step guidance on prescription elements such as dialysate volume, dwell time, number of exchanges, glucose concentration, and the use of icodextrin. Special attention is given to differences between CAPD and APD, highlighting the strengths and limitations of each approach in terms of clearance, ultrafiltration, and patient lifestyle. By combining theoretical background with clinical examples, this teaching resource aims to bridge the gap between guideline recommendations and bedside practice. Ultimately, the article seeks to empower clinicians to prescribe PD with confidence, improve patient-centered decision-making, and foster broader adoption of this therapy, which remains underutilized despite its well-documented clinical and social benefits.

## Introduction

This article aims to review peritoneal dialysis (PD) prescription practices, with a specific focus on automated peritoneal dialysis (APD), and to explore potential strategies to optimize therapy. This discussion takes place in a context where PD has faced significant challenges in maintaining its presence in some countries, with a penetration rate below 5% in Brazil^1^. As a consequence, theoretical knowledge and experience in prescribing this modality—particularly among younger nephrologists—remain limited.

### From Kt/V to Contemporary Approaches

For more than two decades, Kt/V served as the cornerstone of PD prescription, with weekly targets historically ranging from 2.1 to 1.7^2,3^. However, in 2020, the International Society for Peritoneal Dialysis (ISPD) formally redefined the concept of dialysis adequacy^4^. Kt/V, which had already been increasingly questioned in prior years, was officially deemphasized in favor of a more comprehensive approach centered on clinical outcomes, laboratory parameters, and patient quality of life. Accordingly, this article aligns with the current ISPD guidelines and does not address Kt/V as part of its recommendations^4^.

## What Factors Guide the Choice of Peritoneal Dialysis Modality?

There are several ways to prescribe peritoneal dialysis (PD), and the choice of modality should be individualized according to the patient’s clinical profile, residual kidney function, and lifestyle^4,5,6^. Historically, the peritoneal equilibration test (PET) has been used to guide these decisions^7^. The PET evaluates the transport properties of the peritoneal membrane by measuring how quickly solutes such as creatinine and glucose equilibrate between the blood and the dialysis solution during a standardized dwell.

Based on the PET, patients are classified into four categories: high transporters, who absorb glucose and equilibrate solutes very rapidly; low transporters, who exchange solutes more slowly and maintain osmotic gradients for longer; and the intermediate groups, high-average and low-average transporters^7,8^. This classification has important implications for solute clearance and ultrafiltration. High transporters often clear solutes efficiently but may struggle with fluid removal due to rapid glucose absorption, while low transporters may achieve sustained ultrafiltration but require longer dwells to reach adequate solute clearance^9,10^.

Traditionally, these findings influenced the choice of modality. APD was frequently recommended for high transporters, as multiple shorter nighttime exchanges take advantage of rapid solute kinetics while reducing the risk of fluid overload^6^. CAPD was considered more suitable for low transporters, since its longer daytime dwells allow sufficient contact time for solute clearance^11^. However, most patients fall into the intermediate groups, for whom either modality can be effective, particularly if residual renal function is preserved^3,6^.

In modern practice, the decision between APD and CAPD is no longer driven primarily by PET results. Instead, it is guided by patient preference and lifestyle^4,6^. APD may be preferred by younger or employed patients, who may value having free daytime hours, whereas CAPD may appeal to those who prefer independence from machines and a simpler daily routine. PET remains valuable, but rather than dictating the modality, it is now used to fine-tune the prescription, for example, by adjusting dwell times, fill volumes, or selecting the most appropriate solutions^5^.

**Figure 1:**
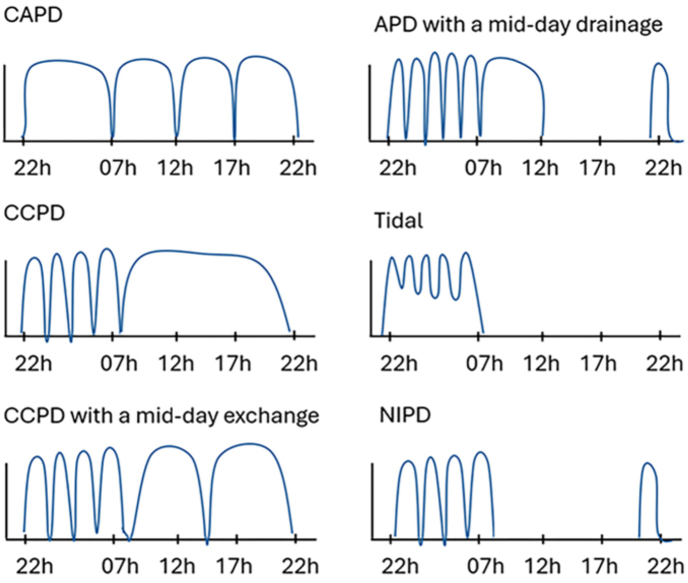
Visual illustration of PD modalities.

Ultimately, modality selection should be the result of a shared decision-making process in which clinical information and patient goals are equally considered. By combining the physiological insights provided by the PET with the patient’s individual circumstances, clinicians can design prescriptions that are both clinically effective and compatible with long-term quality of life^4,5^. Figure 1 illustrates the distribution of exchanges across a 24-hour period for different regimens, while Chart 1 summarizes the key characteristics and recommendations for each modality.

Abbreviations – APD: automated peritoneal dialysis; CAPD: Continuous ambulatory peritoneal dialysis; CCPD: Continuous cycling peritoneal dialysis, Nocturnal intermittent peritoneal dialysis.

**Chart 1:** PD modalities

Modality	Notes
CAPD	It is best suited for patients with a slow peritoneal membrane transport profile. This modality may also be chosen when patients have difficulty using cycler technology; however, in this case, and when the transport profile is fast, adjustments to the prescription will be necessary to avoid ultrafiltration issues that may lead to volume overload. This may involve more stringent daily fluid restrictions and an increased diuretic dosage.
DPA	It is the modality of choice for patients with a fast peritoneal membrane transport profile. It is also preferred when the goal is to allow greater social and occupational activity during the daytime. Patients with a slow transport profile should typically undergo fewer nighttime exchanges, usually between three and four nighttime exchanges.
CCPD	It is the most commonly prescribed modality when there is a need to optimize solute clearance, especially for larger molecules that require longer dwell times. Furthermore, it is particularly useful in patients who have lost their residual urine output. It is performed with 4 to 5 nighttime exchanges and a daytime dwell infusion. The ideal solution currently used for the daytime dwell, when available, is icodextrin. Otherwise, the lowest glucose concentration solution that is still sufficient to maintain positive ultrafiltration should be used.
CCPD + additional daily manual exchange	A daytime exchange is usually necessary when the patient cannot tolerate long dwell times with glucose-based solutions, as their membrane profile allows significant glucose reabsorption, leading to fluid gain throughout the day—especially in settings where icodextrin is not available. It generally places a significant burden on the patient and should be prescribed only when necessary.
NIPD	Particularly useful in patients with good residual kidney function and a fast transport membrane profile.
Tidal^*^	It is often prescribed to help manage pain during fluid drainage, particularly in patients who feel discomfort in the lower abdomen caused by the catheter tip, as well as in cases of catheter tip migration. Typically, the complete fill volume is drained only at the end of therapy, allowing less discomfort and less time connected to the cycler during therapy. The details of the tidal modality prescription will be explored in a separate manuscript.

### Continuous Ambulatory Peritoneal Dialysis (CAPD)

CAPD is one of the earliest PD modalities, in use since the 1970s. Although it remains widely available worldwide, its utilization is lower than that of automated peritoneal dialysis (APD), largely due to the greater convenience associated with APD compared with multiple manual exchanges throughout the day.

CAPD prescriptions are generally standardized, comprising three to four daily exchanges based on the patient’s clinical needs. Except in cases of incremental dialysis (not addressed in this article), the peritoneal cavity remains filled continuously over a 24-hour period. The nighttime dwell is adjusted to align with the patient’s usual sleep duration, while daytime exchanges are typically scheduled upon waking, around midday, and in the late afternoon, resulting in an average dwell time of approximately 5–6 hours. Solutions currently available in the country vary in volume, electrolyte composition, and the type of osmotic agent (Table 1).

**Table 1:** Composition of PD solutions in Brazil

Osmotic Agent	Concentration (%)	Volume (Liters)	Ca++ (mEq/L)	Na+ (mEq/L)	Mg++ (mEq/L)	Cl- (mEq/L)	Lactate (mEq/L)
**CAPD**							
Glucose	1.5, 2.5, and 4.25	2	2.5	132	0.5	95	40
Icodextrin	7.5	2	3.5	133	0.5	96	40
**APD**							
Glucose	1.5, 2.5, and 4.25	2 and 6	3.5	132	0.5	96	40
Glucose	1.5, 2.5, and 4.25	2 and 6	2.5	132	0.5	95	40
Icodextrin	7.5	2	3.5	133	0.5	96	40

### Automated Peritoneal Dialysis (APD)

The optimal parameters for prescribing PD volume involve a careful balance between fill volume, dwell time, and the patient’s body surface area (BSA) to achieve effective solute clearance and fluid management. Several key considerations are required, ranging from the patient’s peritoneal membrane permeability and the available dialysis solutions to the individual’s personal needs. In general, longer dwell times in PD promote solute clearance through diffusion from the capillaries into the peritoneal cavity. However, they may reduce ultrafiltration, since the osmotic agent (typically glucose) also diffuses in the opposite direction— from the peritoneal cavity into the capillaries— diminishing the osmotic gradient over time. This balance between solute clearance and ultrafiltration is individualized, relies on peritoneal membrane characteristics, and is essential for optimizing treatment. The peritoneal equilibration test is the key tool for assessing a patient’s membrane profile and will be discussed in detail in a dedicated manuscript.

## APD was Selected: What Parameters Should be Considered, and How can they be Adjusted?

### Cycler Parameters

The cycler contains several parameters that guide the prescription of PD. These settings determine the efficiency of solute clearance, ultrafiltration, and overall treatment adequacy (Table 2).

**Table 2:** Key cycler settings and recommended ranges

Parameter	Suggested values	Notes
Mode	Standard	The standard mode is for fill volumes over 1000 mL; “Low Fill Mode” is indicated for volumes ranging from 60 to 1000 mL and is usually applied to pediatric patients.
Therapy time	9h (7–10h)	Ranges from 10 min to 48 hours, in 10-minute increments. Extreme values may compromise both patient adherence to treatment and solute clearance.
Fill volume Last fill volume	2 L (1.25–1.5 L/BSA)	In patients with abdominal or inguinal hernias, or those at high risk for developing them, it may be necessary to reduce the dwell volume.
Dextrose	1.5%, 2.5%, 4.25%, or icodextrin 7.5%	It should be chosen according to the patient’s ultrafiltration needs and volume status.
Total Volume	10 L (8–12 L)	Overall volume of fluid utilized for the therapy. It can be set to up to 80,000 mL. Extreme values should be managed cautiously to avoid compromising solute clearance and/or patient adherence.
Number of exchanges	3 to 5	A lower number of exchanges is recommended for patients with lower D/P ratios. Conversely, a higher number is recommended for those with higher D/P values. This parameter is automatically calculated by the cycler.
Smart dwell	Yes or No	Automatically modifies the dwell times to ensure the treatment is completed within the prescribed therapy duration. If set to “No,” the dwell times remain unchanged.
Minimum drainage volume	80% (60 to 125%)^*^	Lower values reduce the number of alarms but increase the risk of excessive intra-abdominal fluid accumulation.

Notes – *Values above 100% are used only for patients with significant ascites production due to advanced liver disease, in whom the goal is to avoid excessive fluid loss during PD.

#### Therapy time

The total therapy time should be adjusted by considering the need for solute clearance and ultrafiltration while never neglecting the patient’s quality of life. For most individuals who rely on peritoneal dialysis, it is possible to tailor the prescription to fit their sleep schedule without requiring them to spend excessively long periods connected to the cycler.

Total therapy time is directly related to dwell time and is therefore an important factor in solute clearance and in maintaining the osmotic gradient. This parameter is usually adjusted to remain between 8 and 9 hours. Shorter durations may compromise solute removal, while longer durations can negatively affect quality of life and treatment adherence. It is essential to always consider the patient’s residual kidney function (RKF) when deciding on therapy time and adjusting it at the first signs of RKF reduction. Table 3 summarizes the expected dwell time in a standard prescription for total therapy times between 6 and 12 hours and for 4 to 6 exchanges per night.

**Table 3:** Approximately dwell-time (in minutes) according to total therapy time and number of exchanges for a PD prescription with 2 L per exchange

Therapy time (h)	Number of exchanges		
	Four	Five	Six
6	64	46	34
7	79	58	44
8	94	70	54
9	109	82	64
10	124	94	74
11	138	106	84
12	153	118	94

#### Fill volume

The fill volume is crucial for maximizing solute clearance and fluid removal and, consequently, is a parameter that must be carefully defined in the prescription. It is important to highlight that this efficiency increases linearly only up to a certain point, beyond which an undue rise in intra-abdominal pressure can impair intraperitoneal blood flow, thereby reducing the efficiency of transmembrane diffusion and convection^9^.

In adults, the infusion volume is usually calculated with less precision than in children, and it is common—albeit inadequate—to prescribe the same 2-L volume for nearly all patients. Ideally, to achieve the best possible outcome, therapy should always be individualized, using an infusion volume of approximately 25 to 35 mL/kg of ideal body weight per 1.73 m^2^. An alternative way to perform this calculation is by using 1.25–1.50 L per square meter of body surface area (calculated using the traditional Du Bois formula)^12^. However, in specific cases—particularly when there is a concern about abdominal hernias or daytime symptoms of abdominal distension—it may be appropriate to prescribe a slightly lower daytime dwell volume compared to the nocturnal exchanges. This adjustment is important because intraperitoneal hydrostatic pressure can vary substantially, typically ranging from 5 to 20 mmHg depending on patient positioning, with lower pressures in the supine position and higher pressures in the upright posture^9^.

#### Last fill and last fill volume

The first parameter determines the prescribed dialysis modality—either CCPD (when set to “Yes”) or NIPD (when set to “No”). When the “Last fill” parameter is set to “Yes,” it is necessary to insert the parameter “Last fill volume.” This is the volume of the last infusion and can be calculated similarly to the volumes used during the nighttime exchanges on the cycler.

#### Dextrose

This option appears only when “Last Fill” is set to “Yes”, so it is used only for the CCPD modality. It instructs the cycler software to use a dialysis solution different from the bags used during the nighttime exchanges for the following long daytime dwell. The cycler setup will use three bags instead of the usual two, and this is the parameter used to prescribe icodextrin 7.5%.

The primary objective of using a third bag for the long dwell is to minimize the risk of osmotic gradient dissipation during the prolonged dwell period. This risk arises when the dextrose option is set to “No”, causing the long dwell to be performed with the hypertonic glucose solution used during the previous night, which typically has a lower glucose concentration. This approach is especially important in patients with fast peritoneal transport rates, as they are more likely to lose the osmotic gradient, often leading to reduced ultrafiltration and fluid overload.

In developing countries, glucose-based PD solutions remain the most used for long dwell times, with the concentration tailored to each patient according to the ultrafiltration obtained. Although the 2.5% concentration is generally preferred due to lower glucose exposure, occasionally 4.25% may be required, especially in anuric fast transporters^13,14^.

#### Icodextrin

Icodextrin, a high–molecular weight glucose polymer, has become a key alternative for the long dwell exchange in PD. Its sustained osmotic effect provides greater ultrafiltration compared with glucose-based solutions, as the osmotic gradient is not rapidly dissipated by absorption. This property enables more effective fluid management during the long dwell, which is particularly valuable for anuric patients, fast transporters, and those requiring stable ultrafiltration to prevent chronic fluid overload^15^.

These results carry important clinical implications. The frequent use of hypertonic 4.25% glucose to achieve adequate ultrafiltration is associated with long-term deleterious effects on the peritoneal membrane, including fibrosis, angiogenesis, and increased permeability. In this context, icodextrin emerges as a more biocompatible solution that reduces daily glucose exposure and helps preserve the peritoneal membrane^11,13,14,16,17,18^.

From a practical standpoint, 7.5% icodextrin should be used for the long dwell because of its sustained osmotic effect. In CAPD, this corresponds to the overnight exchange, while in CCPD, it is most appropriately prescribed for the daytime dwell. This strategy ensures more effective fluid removal during the longest dwell period and helps optimize therapy outcomes.

#### Total volume

The concept of “total therapy volume” is straightforward and refers to the cumulative volume of dialysis solution infused into the peritoneal cavity over a 24-hour period. When the appropriate fill volume per exchange—outlined in the previous sections—is respected, total volume has a direct relationship with solute clearance and ultrafiltration. It is essential to reinforce that the treatment target is no longer the Kt/V, which has proven to be a limited parameter. Instead, a more comprehensive approach has been adopted, incorporating a range of clinical, metabolic, and quality-of-life indicators^4,5^.

The prescribed total volume must be tailored to the patient’s body size, peritoneal membrane characteristics (as determined by the peritoneal equilibration test), and residual renal function. Typically, in CAPD, the total volume usually ranges from 6 to 8 L per day, corresponding to 3 to 4 exchanges of 2 L. In contrast, APD allows for greater variability due to broader prescription options, with most regimens ranging from 8 to 12 L. Although it is possible to prescribe smaller or larger volumes, these are exceptions. The risk of prescribing an inadequate dialysis dose is considerable, as will be discussed in the following section. This last consideration refers specifically to non-incremental PD prescriptions, since such prescriptions are fully appropriate for incremental PD.

The total therapy volume can be adjusted in two different ways: by increasing the number of exchanges or by increasing the fill volume per exchange. Assuming the total therapy time remains the same, the effect on solute clearance differs between the two strategies. In general, the second one—increasing the fill volume—is usually more effective in achieving greater absolute solute removal than increasing the number of exchanges. Increasing the number of exchanges typically enhances ultrafiltration but may result in a relatively smaller improvement, or even a reduction (depending on the number of exchanges increased), in solute clearance. Conversely, increasing the fill volume tends to optimize solute clearance, with only a modest effect on ultrafiltration.

In this context, the introduction of the flow curve concept in the next section will help to prevent a common mistake seen in clinical practice: excessively increasing the number of exchanges, in an attempt to enhance solute clearance, may, paradoxically, lead to the opposite outcome.

#### Number of exchanges

In automated peritoneal dialysis, the cycler does not allow the user to directly select the number of exchanges. Instead, this value is automatically calculated as the total prescribed therapy volume divided by the fill volume per exchange. For example, prescribing 10 L of total dialysate with a 2 L fill volume results in 5 exchanges. If the fill volume is increased to 2.5 L, the number of exchanges decreases to 4.

This mechanism has important clinical implications. Larger fill volumes reduce the number of exchanges and extend the dwell time of each cycle, which may enhance solute and sodium clearance, provided that the total therapy volume is kept constant. Conversely, smaller fill volumes increase the number of exchanges but shorten the effective dwell time, potentially limiting diffusion and convective transport. Thus, careful adjustment of the fill volume is essential to balance dialysis efficiency, fluid removal, and patient tolerance.

**Figure 2:**
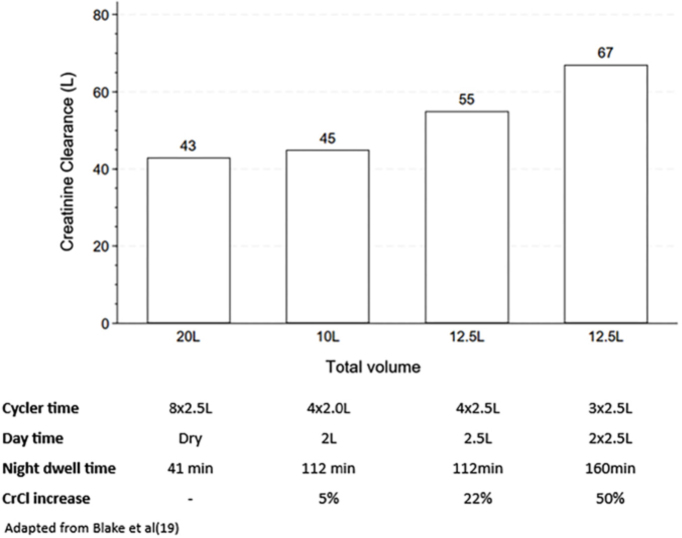
Visual Concept of the peritoneal dialysis flow curve.

A standard chronic PD prescription typically involves 3 to 5 nighttime exchanges. Exceeding 5 exchanges within an 8-to 9-hour therapy period is considered one of the most common errors in PD prescription. Each exchange comprises three distinct phases—infusion, dwell, and drainage. During infusion, and particularly during drainage, there are intervals in which solute diffusion does not occur, representing periods of ineffective dialysis (Figure 2). The higher the number of exchanges within a fixed therapy duration, the greater the cumulative time lost to these non-diffusive phases. Consequently, although the total dialysate volume may increase, the efficiency of solute clearance per unit of time decreases. It should be noted that this principle does not apply to tidal PD, where part of the dialysate is intentionally left in the cavity to minimize “dead time”; this modality will be discussed in detail in a separate article.

Abbreviation – CrCl: creatinine clearance.

In addition to the time lost during infusion and drainage, it is essential to understand that the kinetics of water and sodium transport in peritoneal dialysis are not identical. At the beginning of the dwell, most ultrafiltration occurs through aquaporin-1 (AQP1) water channels, resulting in free water removal without accompanying sodium. This mechanism contributes to achieving negative fluid balance but does not correct extracellular sodium excess. Effective sodium clearance depends primarily on convective transport through the small pores of the peritoneal membrane, where water and solutes are removed together. The extent of this coupled transport is strongly influenced by the volume of dialysate instilled, which determines the “wetted” peritoneal surface area, and by the dwell duration, which allows sufficient time for diffusion and convection to occur.

Clinical and experimental studies demonstrate that short dwells—typically less than 60 to 90 minutes— are inadequate for effective sodium and small solute removal, since the osmotic gradient dissipates before sufficient equilibration has taken place^5,10^. In practice, this means that prescriptions with too many rapid cycles within a fixed therapy period risk delivering suboptimal sodium clearance, even if total ultrafiltration appears adequate. Ensuring a minimum effective dwell time in each exchange is therefore critical to optimize both volume management and metabolic control while still balancing the need for patient comfort and adherence.

**Figure 3:**
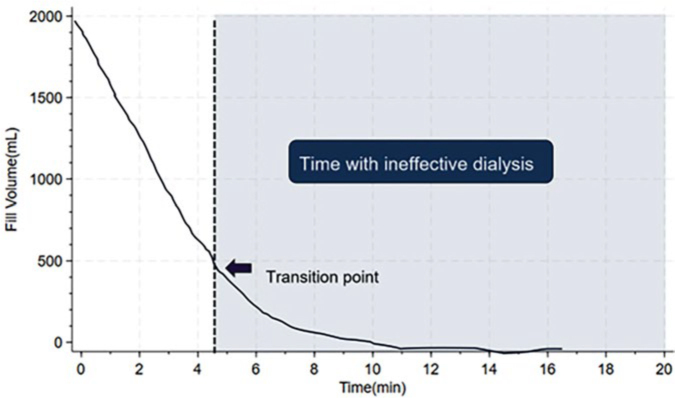
Impact of increasing fill volume in contrast to the number of exchanges in terms of creatinine.

The impact of prescription strategy on solute clearance is better illustrated when comparing changes in fill volume rather than simply increasing the number of exchanges. As shown in Figure 3, studies have demonstrated that higher fill volumes can markedly enhance the effective peritoneal surface area and, consequently, solute transport^8,12^. For example, a CCPD regimen using a total of 12.5 L, supplemented by one daytime manual exchange, achieved nearly a 50% higher creatinine clearance than an NIPD prescription using 20 L of dialysate^9^. This highlights that optimizing fill volume and dwell strategy can yield superior solute removal despite a lower overall dialysate volume, emphasizing the importance of tailoring prescriptions to patient size and peritoneal transport characteristics.

#### Smart dwell

**Figure 4:**
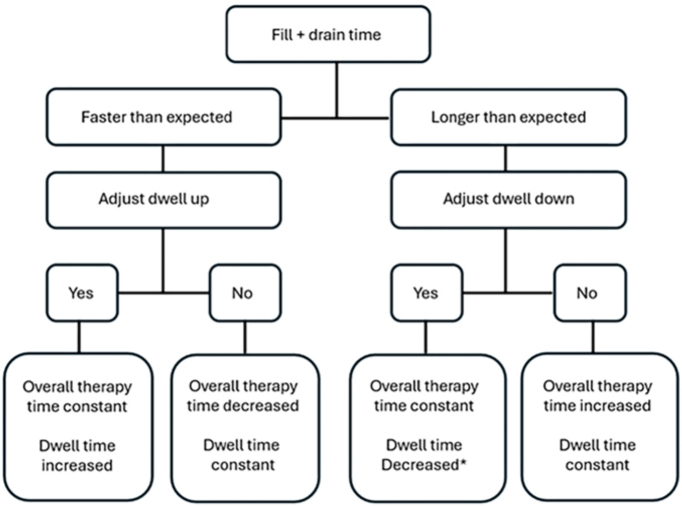
Smart dwell adjustment process in automated peritoneal dialysis.

This parameter defines how the dwell time is managed during APD (Figure 4). It can be set to work automatically, with the cycler adjusting each dwell, or manually, with the dwell time fixed according to the prescription. The main goal is to ensure that the entire therapy fits within the scheduled treatment window, regardless of variations in infusion or drainage speed.

When the option is set to “Yes,” the cycler automatically modifies the dwell time. If drainage or infusion occurs faster than expected, the machine adds more dwell time to keep the therapy on schedule. Conversely, if infusion or drainage is slower, it subtracts minutes from the dwell so that the treatment still finishes within the programmed duration. In this mode, the patient or staff does not need to make manual adjustments.

When the option is set to “No,” the cycler stops making automatic corrections. Instead, two additional settings become available: “Increase dwell time” and “Decrease dwell time.” Each of these can be individually enabled or disabled. This allows greater flexibility but also requires more attention, since inappropriate use can change how effectively the dialysis prescription is delivered.

If “Increase dwell time” is enabled, the cycler will automatically extend the dwell whenever infusion or drainage is completed ahead of schedule. This ensures that the total therapy time remains constant. If “Decrease dwell time” is enabled, the cycler shortens the dwell when infusion or drainage is slower than expected. While this helps the treatment finish on time, it reduces the contact time between dialysate and the peritoneal membrane, which may compromise solute clearance—especially for larger molecules. For this reason, decreasing dwell time should be applied cautiously and only when clinically justified.

#### Manual drainage

At the end of an APD session, it is common for the cycler to leave a small amount of dialysate inside the peritoneal cavity. This residual volume is usually unintentional and results from variations in drainage flow, patient position, or technical limitations of the machine. Although typically modest, the residual volume represents fluid that has not been removed and may contribute to discomfort, abdominal fullness, or suboptimal ultrafiltration.

To address this issue, a final manual drainage can be performed. This procedure consists of connecting the patient to a manual exchange set immediately after the cycler has completed the programmed therapy, allowing the remaining fluid to be drained by gravity. In many cases, this additional step removes several hundred milliliters of dialysate that would otherwise remain in the peritoneal cavity.

The clinical importance of final manual drainage varies. For some patients, the residual volume may be negligible and without consequence. For others, particularly those with limited ultrafiltration capacity or symptoms of abdominal distension, the additional drainage can improve comfort, optimize fluid balance, and ensure a more accurate assessment of net ultrafiltration. It can also reduce the risk of confusion in monitoring, since unrecognized residual dialysate may interfere with the interpretation of drainage volumes recorded by the cycler.

For these reasons, final manual drainage should be considered whenever repeated or significant residual volumes are observed at the end of therapy. Training patients and caregivers on how to perform this step safely can enhance the effectiveness of treatment while at the same time avoiding unnecessary procedures in cases where the residual volume is minimal and clinically irrelevant.

## Conclusion

Peritoneal dialysis represents an effective and patient-centered modality of kidney replacement therapy, but its success depends on a thoughtful and individualized prescription strategy, particularly at the time of initiation. The preservation of residual renal function, careful adjustment of fill volume and dwell duration, and the avoidance of unnecessary exchanges are fundamental principles that directly influence patient outcomes.

## Data Availability

No new data were generated or analyzed in this study.
