# Supplementary material for: Current approaches to prescription and optimization of peritoneal dialysis: a practical review
Source: J Bras Nefrol. 2026 Jun 12;48(2):e20250288. doi: 10.1590/2175-8239-JBN-2025-0288en (PMC13262985; doi:10.1590/2175-8239-JBN-2025-0288en)
Supplement: Translation

# Translation: Abordagens atuais para a prescrição e otimização da diálise peritoneal: uma revisão prática

**DOI:** 10.1590/2175-8239-JBN-2025-0288pt

**Authors:** Thyago Proença de Moraes, Caio Pellizzari, Paulo Ricardo Gessolo Lins, Viviane Calice-Silva

**Affiliations:** 1 Pontifícia Universidade Católica do Paraná, Curitiba, PR, Brasil.; 2 Universidade Federal de São Paulo, São Paulo, SP, Brasil.; 3 Fundação Pró-rim, Departamento de Pesquisa, Joinville, SC, Brasil.; 4 Universidade da Região de Joinville, Faculdade de Medicina, Joinville, SC, Brasil.

**Keywords:** Prescrições, Função Renal Residual, Ultrafiltração, Icodextrina, Diálise Peritoneal Automatizada, Diálise Peritoneal Ambulatorial Contínua

## Abstract

A diálise peritoneal (DP) é uma forma estabelecida de terapia renal substitutiva que oferece aos pacientes a possibilidade de tratamento domiciliar, com significativa flexibilidade e autonomia. Apesar de suas vantagens, muitos médicos em formação e em início de prática clínica sentem-se inseguros ao prescrever DP, diante dos aspectos técnicos, dos ajustes individualizados e da variedade de modalidades disponíveis. Este manuscrito fornece uma visão geral prática e didática sobre como prescrever DP, com foco tanto na diálise peritoneal ambulatorial contínua (CAPD) quanto na diálise peritoneal automatizada (APD). A discussão inicia-se com os princípios fundamentais do transporte de solutos e de fluidos, seguida por orientações passo a passo sobre os principais elementos da prescrição, como volume do dialisato, tempo de permanência, número de trocas, concentração de glicose e uso de icodextrina. Atenção especial é dada às diferenças entre CAPD e APD, destacando os pontos fortes e as limitações de cada abordagem em termos de depuração, ultrafiltração e estilo de vida do paciente. Ao combinar embasamento teórico com exemplos clínicos, este material didático busca reduzir a distância entre as recomendações de diretrizes e a prática à beira do leito. Em última análise, o artigo pretende capacitar os clínicos a prescrever DP com maior confiança, melhorar a tomada de decisão centrada no paciente e fomentar a adoção mais ampla dessa terapia, que permanece subutilizada, apesar de seus benefícios clínicos e sociais bem documentados.

## Introdução

Este artigo tem como objetivo revisar as práticas de prescrição em diálise peritoneal (DP), com foco específico na diálise peritoneal automatizada (APD), e explorar estratégias potenciais para otimizar a terapia. Essa discussão ocorre em um contexto no qual a DP tem enfrentado desafios importantes para manter sua presença em alguns países, com taxa de penetração inferior a 5% no Brasil^1^. Como consequência, o conhecimento teórico e a experiência na prescrição dessa modalidade — particularmente entre nefrologistas mais jovens — permanecem limitados.

### Do Kt/V às Abordagens Contemporâneas

Por mais de duas décadas, o Kt/V serviu como pilar da prescrição em DP, com metas semanais que historicamente variaram de 2,1 a 1,7^2,3^. No entanto, em 2020, a Sociedade Internacional de Diálise Peritoneal (ISPD) redefiniu formalmente o conceito de adequação dialítica^4^. O Kt/V, que já vinha sendo cada vez mais questionado nos anos anteriores, foi oficialmente despriorizado em favor de uma abordagem mais abrangente, centrada em desfechos clínicos, parâmetros laboratoriais e qualidade de vida do paciente.

De acordo com essa nova perspectiva, o presente artigo está alinhado às diretrizes atuais da ISPD e, portanto, não aborda o Kt/V como componente central de suas recomendações^4^.

## Quais Fatores Orientam a Escolha da Modalidade de Diálise Peritoneal?

Existem diversas formas de prescrever DP, e a escolha da modalidade deve ser individualizada de acordo com o perfil clínico do paciente, a função renal residual e o estilo de vida ^4,5,6^. Historicamente, o teste de equilíbrio peritoneal (PET) foi utilizado para orientar essas decisões^7^. O PET avalia as propriedades de transporte da membrana peritoneal ao medir a velocidade com que solutos, como creatinina e glicose, se equilibram entre o sangue e a solução de diálise durante um período padronizado de permanência.

Com base no PET, os pacientes são classificados em quatro categorias: transportadores rápidos, que absorvem glicose e equilibram solutos muito rapidamente; transportadores lentos, que realizam a troca de solutos de forma mais lenta e mantêm o gradiente osmótico por mais tempo; e os transportadores intermediários, classificados como médio-rápidos e médio-lentos^7,8^. Essa classificação tem implicações importantes para a depuração de solutos e para a ultrafiltração. Transportadores rápidos frequentemente apresentam boa depuração de solutos, mas podem ter dificuldade na remoção de líquidos devido à rápida absorção de glicose, enquanto transportadores lentos podem alcançar ultrafiltração sustentada, porém necessitam de tempos de permanência mais longos para obter depuração adequada^9,10^.

Tradicionalmente, esses achados influenciavam a escolha da modalidade. A APD é frequentemente recomendada para transportadores rápidos, uma vez que múltiplas trocas noturnas mais curtas aproveitam a cinética rápida de solutos, ao mesmo tempo em que reduzem o risco de sobrecarga volêmica^6^. A CAPD é considerada mais adequada para transportadores lentos, pois seus tempos de permanência diurnos mais longos permitem contato suficiente para a depuração de solutos^11^. No entanto, a maioria dos pacientes pertence aos grupos intermediários, para os quais ambas as modalidades podem ser eficazes, especialmente quando a função renal residual está preservada^3,6^.

Na prática moderna, a decisão entre APD e CAPD não é mais guiada primariamente pelos resultados do PET. Em vez disso, é orientada pelas preferências e pelo estilo de vida do paciente^4,6^. A APD pode ser preferida por pacientes mais jovens ou que trabalham, que valorizam ter o dia livre, enquanto a CAPD pode atrair aqueles que preferem independência de máquinas e uma rotina diária mais simples. O PET continua sendo uma ferramenta valiosa, mas, em vez de ditar a modalidade, passou a ser utilizado para ajustar finamente a prescrição, por exemplo, ao modificar tempos de permanência, volumes de infusão ou ao selecionar as soluções mais adequadas^5^.

Em última análise, a seleção da modalidade deve ser resultado de um processo de decisão compartilhada, no qual as informações clínicas e os objetivos do paciente são igualmente considerados. Ao combinar os conhecimentos fisiológicos fornecidos pelo PET com as circunstâncias individuais do paciente, os clínicos podem elaborar prescrições que sejam ao mesmo tempo clinicamente eficazes e compatíveis com a qualidade de vida a longo prazo^4,5^. A Figura 1 ilustra a distribuição das trocas ao longo de 24 horas para diferentes regimes, enquanto a Quadro 1 resume as principais características e recomendações para cada modalidade.

**Figura 1:** Ilustração visual das modalidades de diálise peritoneal.

Abreviaturas – DPA: diálise peritoneal automatizada; DPAC: Diálise peritoneal ambulatorial contínua; DPCC: Diálise peritoneal contínua por cicladora; DPNI: Diálise peritoneal noturna intermitente.

**Quadro 1:** Modalidades de diálise peritoneal

Modalidade	Notas
CAPD	É mais indicada para pacientes com perfil de transporte peritoneal lento. Essa modalidade também pode ser escolhida quando o paciente apresenta dificuldade no uso da tecnologia da cicladora; entretanto, nesse caso — e especialmente quando o perfil de transporte é rápido — serão necessários ajustes na prescrição para evitar problemas de ultrafiltração que podem levar à sobrecarga volêmica. Isso pode envolver restrições mais rigorosas de ingestão hídrica diária e aumento da dose de diuréticos.
DPA	É a modalidade de escolha para pacientes com perfil de transporte peritoneal rápido. Também é preferida quando o objetivo é permitir maior atividade social e ocupacional durante o dia. Pacientes com perfil de transporte lento devem, em geral, realizar menos trocas noturnas, normalmente entre três e quatro.
CCPD	É a modalidade mais comumente prescrita quando há necessidade de otimizar a depuração de solutos, especialmente de moléculas maiores que requerem tempos de permanência mais longos. É particularmente útil em pacientes que perderam o débito urinário residual. Realiza-se com 4 a 5 trocas noturnas e uma infusão de longa permanência durante o dia. A solução ideal atualmente para a longa permanência diurna, quando disponível, é a icodextrina. Caso contrário, deve-se utilizar a menor concentração de glicose que ainda seja suficiente para manter a ultrafiltração positiva.
CCPD ^+^ troca manual diurna adicional	Uma troca diurna geralmente é necessária quando o paciente não tolera longos tempos de permanência com soluções à base de glicose, uma vez que o perfil de membrana permite a reabsorção significativa de glicose, levando ao ganho de líquido ao longo do dia — especialmente em locais onde a icodextrina não está disponível. Essa estratégia impõe, em geral, um ônus significativo ao paciente e deve ser prescrita apenas quando estritamente necessário.
NIPD	Particularmente útil em pacientes com boa função renal residual e perfil de transporte peritoneal rápido.
Tidal^*^	Frequentemente prescrita para auxiliar no manejo da dor durante a drenagem do fluido, especialmente em pacientes que sentem desconforto no abdome inferior causado pela ponta do cateter, bem como em casos de migração da ponta do cateter. Normalmente, o volume total de enchimento é drenado apenas ao final da terapia, permitindo menor desconforto e menor tempo efetivo de drenagem durante o tratamento. Os detalhes da prescrição da modalidade tidal serão explorados em um manuscrito separado.

### Diálise Peritoneal Ambulatorial Contínua (CAPD)

A CAPD é uma das modalidades mais antigas de DP, em uso desde a década de 1970. Embora ainda esteja amplamente disponível em todo o mundo, sua utilização é menor do que a da APD, em grande parte devido à maior conveniência associada à APD, em comparação com as múltiplas trocas manuais ao longo do dia.

As prescrições de CAPD são, em geral, padronizadas, compreendendo três a quatro trocas diárias, de acordo com as necessidades clínicas do paciente. Exceto nos casos de diálise incremental (não abordados neste artigo), a cavidade peritoneal permanece preenchida continuamente ao longo de 24 horas. O tempo de permanência noturno é ajustado para coincidir com a duração habitual do sono do paciente, enquanto as trocas diurnas são tipicamente programadas ao despertar, próximo ao meio-dia e no final da tarde, resultando em um tempo médio de permanência de aproximadamente 5 a 6 horas. As soluções atualmente disponíveis no país variam em volume, composição eletrolítica e tipo de agente osmótico (Tabela 1).

**Tabela 1:** Composição das soluções de diãlise peritoneal no brasil

Agente osmótico	Concentração (%)	Volume (Litros)	Ca++ (mEq/L)	Na+ (mEq/L)	Mg++ (mEq/L)	Cl-(mEq/L)	Lactato (mEq/L)
**CAPD**							
Glicose	1,5; 2,5; e 4,25	2	2,5	132	0,5	95	40
Icodextrina	7,5	2	3,5	133	0,5	96	40
**APD**							
Glicose	1,5; 2,5; e 4,25	2 e 6	3,5	132	0,5	96	40
Glicose	1,5; 2,5; e 4,25	2 e 6	2,5	132	0,5	95	40
Icodextrina	7,5	2	3,5	133	0,5	96	40

### Diálise Peritoneal Automatizada (APD)

Os parâmetros ideais para a prescrição do volume em DP envolvem um equilíbrio cuidadoso entre volume de infusão, tempo de permanência e área de superfície corporal do paciente, de modo a alcançar depuração eficaz de solutos e manejo adequado de fluidos. Diversas considerações são necessárias, desde a permeabilidade da membrana peritoneal do paciente e as soluções de diálise disponíveis até as necessidades individuais.

Em geral, tempos de permanência mais longos na DP favorecem a depuração de solutos por difusão dos capilares para a cavidade peritoneal. No entanto, podem reduzir a ultrafiltração, uma vez que o agente osmótico (tipicamente a glicose) também se difunde no sentido oposto — da cavidade peritoneal para os capilares — diminuindo o gradiente osmótico ao longo do tempo. Esse equilíbrio entre depuração de solutos e ultrafiltração é individualizado, depende das características da membrana peritoneal e é essencial para a otimização do tratamento. O teste de equilíbrio peritoneal é a principal ferramenta para avaliar o perfil da membrana do paciente e será discutido em detalhe em um manuscrito dedicado.

## A APD foi Escolhida: Quais Parâmetros Devem ser Considerados e Como Podem ser Ajustados?

### Parâmetros da Cicladora

A cicladora contém diversos parâmetros que orientam a prescrição da DP. Essas configurações determinam a eficiência da depuração de solutos, da ultrafiltração e da adequação global do tratamento (Tabela 2).

**Tabela 2:** Principais parâmetros da cicladora e faixas recomendadas

Parâmetro	Valores sugeridos	Observações
Modo	Padrão	O modo padrão é utilizado para volumes de infusão acima de 1000 mL; o “Modo de Baixo Volume” é indicado para volumes entre 60 e 1000 mL e é geralmente aplicado em pacientes pediátricos.
Tempo de terapia	9h (7–10h)	Varia de 10 minutos a 48 horas, em incrementos de 10 minutos. Valores extremos podem comprometer tanto a adesão ao tratamento quanto a depuração de solutos.
Volume de infusão		Em pacientes com hérnias abdominais ou inguinais, ou naqueles com alto risco de desenvolvê-las, pode ser necessário reduzir o volume de permanência. Deve ser escolhido de acordo com as necessidades de ultrafiltração e o estado volêmico do paciente.
Volume da última infusão	2 L (1,25–1,5 L/BSA)	Deve ser definido quando a modalidade CCPD é selecionada (longa permanência diurna).
Dextrose	1,5%; 2,5%; 4,25%; ou icodextrina 7,5%	Utilizada na longa permanência quando a opção “Última infusão” está ativada.
Volume total	10 L (8–12 L)	Volume total de fluido utilizado na terapia; pode ser ajustado até 80.000 mL. Valores extremos devem ser manejados com cautela para não comprometer a depuração de solutos e/ou a adesão do paciente.
Número de trocas	3 a 5	Recomenda-se menor número de trocas para pacientes com menores razões D/P e maior número para aqueles com maiores valores de D/P. Esse parâmetro é calculado automaticamente pela cicladora.
Permanência inteligente	Sim ou Não	Modifica automaticamente os tempos de permanência para garantir que o tratamento seja concluído no tempo prescrito. Quando definido como “Não”, os tempos de permanência permanecem inalterados.
Volume mínimo de drenagem	80% (60 a 125%)^*^	Valores mais baixos reduzem o número de alarmes, mas aumentam o risco de acúmulo excessivo de fluido intra-abdominal.

Nota – ^*^Valores acima de 100% são utilizados apenas em pacientes com produção significativa de ascite devido à doença hepática avançada, nos quais o objetivo é evitar perda excessiva de fluidos durante a DP.

#### Tempo de terapia

O tempo total de terapia deve ser ajustado levando em consideração a necessidade de depuração de solutos e ultrafiltração, sem jamais esquecer a qualidade de vida do paciente. Para a maioria dos indivíduos em DP, é possível adaptar a prescrição ao seu horário de sono, sem impor períodos excessivamente longos conectados à cicladora.

Esse parâmetro está diretamente relacionado ao tempo de permanência e, portanto, é um fator importante tanto para a depuração de solutos quanto para a manutenção do gradiente osmótico. Em geral, é ajustado para permanecer entre 8 e 9 horas na cavidade abdominal. Durações mais curtas podem comprometer a remoção de solutos, enquanto períodos mais longos podem afetar negativamente a qualidade de vida e a adesão ao tratamento. É fundamental sempre considerar a função renal residual (FRR) ao definir o tempo de terapia e ajustálo aos primeiros sinais de sua redução.

A Tabela 3 resume o tempo de permanência esperado em uma prescrição padrão para tempos totais de terapia entre 6 e 12 horas e para 4 a 6 trocas por noite.

**Tabela 3:** Tempo aproximado de permanência (em minutos) de acordo com o tempo total de terapia e o número de trocas para uma Prescrição de DP com 2 L por troca

Tempo de terapia (h)	Número de trocas		
	Quatro	Cinco	Seis
6	64	46	34
7	79	58	44
8	94	70	54
9	109	82	64
10	124	94	74
11	138	106	84
12	153	118	94

#### Volume de infusão

O volume de infusão é fundamental para maximizar a depuração de solutos e a remoção de fluidos e, consequentemente, é um parâmetro que deve ser cuidadosamente definido na prescrição. É importante destacar que essa eficiência aumenta de forma linear apenas até certo ponto, a partir do qual um aumento excessivo da pressão intra-abdominal pode comprometer o fluxo sanguíneo intraperitoneal, reduzindo a eficiência da difusão e da convecção transmembrana^9^.

Em adultos, o volume de infusão é geralmente calculado com menor precisão do que em crianças, sendo comum — ainda que inadequado — prescrever o mesmo volume de 2 L para quase todos os pacientes. Idealmente, para alcançar o melhor desfecho possível, a terapia deve ser sempre individualizada, utilizando-se um volume de infusão de aproximadamente 25 a 35 mL/kg de peso corporal ideal por 1,73 m^2^.

Uma forma alternativa de realizar esse cálculo é utilizar 1,25 a 1,50 L por metro quadrado de área de superfície corporal, calculada pela fórmula tradicional de Du Bois^12^. No entanto, em situações específicas — particularmente quando há preocupação com hérnias abdominais ou sintomas diurnos de distensão — pode ser apropriado prescrever um volume de permanência diurno ligeiramente menor em comparação com as trocas noturnas. Esse ajuste é relevante porque a pressão hidrostática intraperitoneal pode variar substancialmente, geralmente entre 5 e 20 mmHg, dependendo da posição do paciente, com pressões menores em decúbito e maiores na posição ortostática^9^.

#### Última infusão e volume da última infusão

O primeiro parâmetro determina a modalidade prescrita — CCPD (quando ajustado como “Sim”) ou NIPD (quando ajustado como “Não”). Quando o parâmetro “Última infusão” é definido como “Sim”, torna-se necessário inserir o “Volume da última infusão”, que corresponde ao volume da infusão final e pode ser calculado de forma semelhante aos volumes utilizados durante as trocas noturnas na cicladora.

#### Dextrose

Esta opção aparece apenas quando o parâmetro “Última infusão” está definido como “Sim”, sendo utilizada exclusivamente na modalidade CCPD. Ela instrui o software da cicladora a utilizar uma solução de diálise diferente daquelas usadas durante as trocas noturnas para a longa permanência diurna. Nesse caso, a cicladora passa a utilizar três bolsas, em vez das duas habituais, sendo esse o parâmetro utilizado para prescrever icodextrina 7,5%.

O principal objetivo do uso de uma terceira bolsa para o longo tempo de permanência é minimizar o risco de dissipação do gradiente osmótico durante esse período prolongado. Esse risco ocorre quando a opção “Dextrose” é definida como “Não”, fazendo com que a longa permanência seja realizada com a mesma solução de glicose utilizada na noite anterior, que geralmente apresenta menor concentração de glicose. Essa abordagem é particularmente importante em pacientes com transporte peritoneal rápido, os quais são mais propensos à perda precoce do gradiente osmótico, frequentemente resultando em ultrafiltração reduzida e sobrecarga volêmica.

Em países em desenvolvimento, as soluções à base de glicose ainda são as mais utilizadas para tempos de permanência prolongados, com a concentração ajustada de acordo com a ultrafiltração obtida. Embora a concentração de 2,5% seja geralmente preferida por resultar em menor exposição à glicose, ocasionalmente pode ser necessária a solução a 4,25%, especialmente em pacientes anúricos e com transporte rápido^13,14^.

#### Icodextrin

A icodextrina, um polímero de glicose de alto peso molecular, tornou-se uma alternativa fundamental para uso na longa permanência. Seu efeito osmótico sustentado proporciona maior ultrafiltração em comparação às soluções à base de glicose, uma vez que o gradiente osmótico não é rapidamente dissipado por absorção. Essa propriedade permite um manejo mais eficaz de fluidos durante a longa permanência, sendo particularmente valiosa para pacientes anúricos, transportadores rápidos e aqueles que necessitam de ultrafiltração estável para prevenir sobrecarga hídrica crônica^15^.

Esses resultados têm implicações clínicas importantes. O uso frequente de glicose hipertônica a 4,25% para alcançar ultrafiltração adequada está associado a efeitos deletérios de longo prazo sobre a membrana peritoneal, incluindo fibrose, neoangiogênese e aumento da permeabilidade capilar. Nesse contexto, a icodextrina surge como uma solução mais biocompatível, que reduz a exposição diária à glicose e ajuda a preservar a membrana peritoneal^11,13,14,16,17,18^.

Na prática clínica, a icodextrina 7,5% deve ser utilizada sempre na longa permanência devido ao seu efeito osmótico sustentado mencionado anteriormente. Na CAPD, isso corresponde à troca noturna; na CCPD, é mais apropriadamente prescrita para o tempo de permanência diurno. Essa estratégia assegura uma remoção de fluidos mais eficaz durante o período mais prolongado e contribui para a otimização dos desfechos terapêuticos.

#### Volume total

O conceito de volume total de terapia é simples e refere-se ao volume cumulativo de solução de diálise infundido na cavidade peritoneal ao longo de 24 horas. Quando o volume de infusão por troca — conforme descrito nas seções anteriores — é respeitado, o volume total apresenta relação direta com a depuração de solutos e a ultrafiltração.

É importante reforçar que o alvo do tratamento não é mais o Kt/V, que se mostrou um parâmetro limitado. Em seu lugar, foi adotada uma abordagem mais abrangente, incorporando um conjunto de indicadores clínicos, metabólicos e de qualidade de vida^4,5^.

O volume total prescrito deve ser ajustado ao tamanho corporal do paciente, às características da membrana peritoneal (determinadas pelo teste de equilíbrio peritoneal) e à função renal residual. Tipicamente, na CAPD, o volume total varia entre 6 e 8 L por dia, correspondendo a 3 a 4 trocas de 2 L. Em contraste, a APD permite maior variabilidade, com a maioria dos esquemas situandose entre 8 e 12 L. Embora seja possível prescrever volumes menores ou maiores, essas situações são exceções, e o risco de uma dose de diálise inadequada é considerável, conforme será discutido na seção seguinte. Essa última consideração aplicase especificamente a prescrições não incrementais, uma vez que tais esquemas são plenamente apropriados para DP incremental.

O volume total pode ser ajustado de duas formas: aumentando o número de trocas ou aumentando o volume de infusão por troca. Supondo que o tempo total de terapia permaneça o mesmo, o efeito sobre a depuração de solutos difere entre essas estratégias. Em geral, a segunda — aumentar o volume de infusão — é mais eficaz para alcançar maior remoção absoluta de solutos do que aumentar o número de trocas. O aumento do número de trocas tende a intensificar a ultrafiltração, mas pode resultar em melhora relativamente menor, ou até mesmo em redução (dependendo de quantas trocas são adicionadas) da depuração de solutos. Por outro lado, a elevação do volume de infusão tende a otimizar a depuração de solutos, com impacto apenas modesto sobre a ultrafiltração.

Nesse contexto, a introdução do conceito de curva de fluxo na próxima seção ajudará a evitar um erro comum observado na prática clínica: aumentar excessivamente o número de trocas, na tentativa de melhorar a depuração de solutos, pode, paradoxalmente, levar ao efeito oposto.

#### Número de trocas

Na diálise peritoneal automatizada, a cicladora não permite que o usuário selecione diretamente o número de trocas. Em vez disso, esse valor é calculado automaticamente a partir do volume total de dialisato prescrito dividido pelo volume de infusão por troca. Por exemplo, ao prescrever 10 L de volume total com volume de infusão de 2 L, resultam 5 trocas. Se o volume de infusão for aumentado para 2,5 L, o número de trocas diminui para 4.

Esse mecanismo tem implicações clínicas importantes. Volumes de infusão maiores reduzem o número de trocas e prolongam o tempo de permanência de cada ciclo, o que pode melhorar a depuração de solutos e de sódio, desde que o volume total de terapia seja mantido constante. Em contrapartida, volumes menores aumentam o número de trocas, mas encurtam o tempo efetivo de permanência, podendo limitar a difusão e o transporte convectivo. Assim, o ajuste cuidadoso do volume de infusão é essencial para equilibrar a eficiência dialítica, a remoção de fluidos e a tolerabilidade do paciente.

Uma prescrição crônica padrão de DP geralmente envolve 3 a 5 trocas noturnas. Ultrapassar 5 trocas dentro de um período de terapia de 8 a 9 horas é considerado um dos erros mais comuns na prescrição de DP. Cada troca compreende três fases distintas — infusão, permanência e drenagem. Durante a infusão e, especialmente, durante a drenagem, existem intervalos em que não ocorre difusão de solutos, representando períodos de diálise inefetiva (Figura 2). Quanto maior for o número de trocas dentro de um tempo fixo de terapia, maior é o tempo cumulativo perdido nessas fases não difusivas. Consequentemente, embora o volume total de dialisato possa aumentar, a eficiência da depuração de solutos por unidade de tempo diminui. Deve-se ressaltar que esse princípio não se aplica à DP tidal, na qual parte do dialisato é intencionalmente mantida na cavidade para minimizar o “tempo morto”; essa modalidade será discutida em artigo separado.

**Figura 2:** Conceito visual da curva de fluxo da diálise peritoneal.

Abreviatura – CrCl: Clearance de creatinina.

Além do tempo perdido durante a infusão e a drenagem, é essencial compreender que a cinética de transporte de água e sódio na diálise peritoneal não é idêntica. No início da permanência, a maior parte da ultrafiltração ocorre por meio dos canais de água aquaporina-1 (AQP1), resultando em remoção de água livre, sem sódio associado. Esse mecanismo contribui para alcançar um balanço hídrico negativo, mas não corrige o excesso extracelular de sódio. A depuração eficaz de sódio depende principalmente do transporte convectivo através dos pequenos poros da membrana peritoneal, onde água e solutos são removidos conjuntamente.

A extensão desse transporte acoplado é fortemente influenciada pelo volume de dialisato instilado — que determina a área peritoneal “molhada” — e pela duração da permanência, que permite tempo suficiente para que a difusão e a convecção ocorram. Estudos clínicos e experimentais demonstram que permanências curtas — tipicamente inferiores a 60 a 90 minutos — são inadequadas para a remoção eficaz de sódio e pequenos solutos, uma vez que o gradiente osmótico se dissipa antes que ocorra equilibrização suficiente^5,10^. Na prática, isso significa que prescrições com muitos ciclos rápidos dentro de um tempo fixo de terapia correm o risco de fornecer depuração subótima de sódio, mesmo quando a ultrafiltração total parece adequada. Garantir um tempo mínimo efetivo de permanência em cada troca é, portanto, fundamental para otimizar tanto o manejo volêmico quanto o controle metabólico, mantendo o equilíbrio com conforto e adesão do paciente.

O impacto da estratégia de prescrição sobre a depuração de solutos é melhor ilustrado ao se comparar mudanças no volume de infusão, em vez de simplesmente aumentar o número de trocas. Conforme demonstrado na Figura 3, estudos demonstram que volumes de infusão mais elevados podem aumentar de forma significativa a área efetiva de superfície peritoneal e, consequentemente, o transporte de solutos^8,12^. Por exemplo, um regime de CCPD utilizando volume total de 12,5 L, suplementado por uma troca manual diurna, alcançou quase 50% mais depuração de creatinina do que uma prescrição de NIPD com 20 L de dialisato^9^. Isso evidencia que a otimização do volume de infusão e da estratégia de permanência pode proporcionar remoção superior de solutos, mesmo com menor volume total de dialisato, reforçando a importância de individualizar a prescrição de acordo com o tamanho corporal e as características de transporte peritoneal.

**Figura 3:** Impacto do aumento do volume de enchimento em comparação com o número de trocas em termos de depuração de creatinina.

#### Smart dwell

Este parâmetro define como o tempo de permanência é gerenciado durante a APD (Figura 4). Ele pode ser configurado para funcionamento automático, com a cicladora ajustando cada permanência, ou manual, com tempo de permanência fixo conforme a prescrição. O principal objetivo é garantir que toda a terapia se encaixe dentro da janela de tratamento programada, independentemente de variações na velocidade de infusão ou drenagem.

**Figura 4:** Processo de ajuste da permanência inteligente na diálise peritoneal automatizada.

Quando a opção é definida como “Sim”, a cicladora modifica automaticamente o tempo de permanência. Se a drenagem ou a infusão ocorrerem mais rapidamente que o esperado, a máquina adiciona tempo de permanência para assegurar que a terapia siga o cronograma. Por outro lado, se a infusão ou a drenagem forem mais lentas, a cicladora subtrai minutos da permanência para que o tratamento ainda termine dentro do tempo programado.

Quando a opção é definida como “Não”, a cicladora deixa de realizar correções automáticas. Nesse caso, dois ajustes adicionais tornam-se disponíveis: “Aumentar tempo de permanência” e “Reduzir tempo de permanência”. Cada um pode ser ativado ou desativado individualmente. Isso confere maior flexibilidade, mas também exige maior atenção, pois o uso inadequado pode alterar a eficácia da prescrição.

Se “Aumentar tempo de permanência” estiver ativado, a cicladora estenderá automaticamente a permanência sempre que a infusão ou a drenagem forem concluídas antes do previsto, garantindo que o tempo total de terapia permaneça constante.

Se “Reduzir tempo de permanência” estiver ativado, a cicladora encurtará a permanência quando a infusão ou a drenagem forem mais lentas que o esperado. Embora isso ajude a manter o término da sessão no horário programado, reduz o tempo de contato entre o dialisato e a membrana peritoneal, podendo comprometer a depuração de solutos — especialmente de moléculas maiores. Por esse motivo, a redução do tempo de permanência deve ser aplicada com cautela e apenas quando clinicamente justificada.

#### Drenagem manual final

Ao término de uma sessão de APD, é comum que a cicladora deixe um pequeno volume de dialisato na cavidade peritoneal. Esse volume residual é geralmente não intencional e resulta de variações no fluxo de drenagem, na posição do paciente ou de limitações técnicas do equipamento. Embora, em geral, seja modesto, esse volume representa líquido que não foi removido e pode contribuir para desconforto, sensação de plenitude abdominal ou ultrafiltração subótima.

Para lidar com essa situação, pode-se realizar uma “drenagem manual final”. Esse procedimento consiste em conectar o paciente a um equipamento de troca manual imediatamente após a cicladora concluir a terapia programada, permitindo que o líquido remanescente seja drenado por gravidade. Em muitos casos, essa etapa adicional remove várias centenas de mililitros de dialisato que, de outra forma, permaneceriam na cavidade.

A importância clínica da drenagem manual final varia. Para alguns pacientes, o volume residual pode ser irrelevante; para outros — especialmente aqueles com capacidade limitada de ultrafiltração ou sintomas de distensão abdominal — a drenagem adicional pode melhorar o conforto, otimizar o balanço hídrico e garantir uma avaliação mais precisa da ultrafiltração líquida. Também reduz o risco de interpretações equivocadas, uma vez que o dialisato residual não reconhecido pode interferir na leitura dos volumes de drenagem registrados pela cicladora.

Por essas razões, a drenagem manual final deve ser considerada sempre que volumes residuais repetidos ou significativos forem observados ao final da terapia. O treinamento de pacientes e cuidadores para realizar esse procedimento de forma segura pode aumentar a efetividade do tratamento, ao mesmo tempo em que evita intervenções desnecessárias quando o volume residual é mínimo e clinicamente irrelevante.

## Conclusão

A diálise peritoneal representa uma modalidade eficaz e centrada no paciente de terapia renal substitutiva, mas seu sucesso depende de uma estratégia de prescrição criteriosa e individualizada, particularmente no momento da iniciação. A preservação da função renal residual, o ajuste cuidadoso do volume de infusão e do tempo de permanência, e a evitação de trocas desnecessárias são princípios fundamentais que influenciam diretamente os desfechos dos pacientes.

## Data Availability

Nenhum dado novo foi gerado ou analisado neste estudo.
